# Cost-effectiveness of a novel antitachycardia pacing algorithm for the termination of ventricular tachyarrhythmias in the United States

**DOI:** 10.1016/j.hroo.2026.05.015

**Published:** 2026-06-02

**Authors:** Steven Zweibel, Jonathan W. Dukes, Anthony I. Roberts, Reece Holbrook, Julen Monje, Joseph D. Mozingo, Anne B. Curtis

**Affiliations:** 1 Hartford HealthCare Heart and Vascular Institute, Hartford, Connecticut; 2 University of Connecticut School of Medicine, Farmington, Connecticut; 3 Division of electrophysiology, Community Memorial Hospital, Ventura, California; 4 Medtronic, Inc., Mounds View, Minnesota; 5 Medtronic Ibérica, Madrid, Spain; 5 Jacobs School of Medicine and Biomedical Sciences, University at Buffalo, Buffalo, New York

**Keywords:** Antitachycardia pacing, Ventricular tachycardia, Ventricular fibrillation, Implantable cardioverter-defibrillator, Cost-effectiveness

## Abstract

**Background:**

Ventricular tachyarrhythmias are potentially life-threatening arrhythmias commonly managed with implantable cardioverter-defibrillators (ICDs). Conventional antitachycardia pacing (ATP) can reduce ICD shocks, but its effectiveness varies. Intrinsic antitachycardia pacing (iATP) is a novel algorithm designed to optimize ventricular tachycardia termination and minimize shocks.

**Objective:**

The purpose of this study was to evaluate the cost-effectiveness of iATP vs conventional ATP for terminating ventricular tachyarrhythmias in the United States.

**Methods:**

A Markov simulation model was developed to compare lifetime costs, survival, quality-adjusted life-years (QALYs), and incremental cost-effectiveness ratio (ICER) of iATP vs conventional ATP in patients with ICDs. Model inputs were derived from published literature, and analyses were performed from the perspective of an integrated US health care system. Sensitivity analyses assessed the robustness of findings.

**Results:**

Compared with conventional ATP, iATP was associated with additional QALYs (10.51 vs 10.33) and additional lifetime costs ($4014 vs $1673), resulting in a discounted ICER of $13,114 per QALY. The ICER is below the US willingness-to-pay (WTP) threshold ($120,000 per QALY). The finding of cost-effectiveness persisted across all sensitivity analyses.

**Conclusion:**

iATP was associated with clinical benefits and was determined to be a highly cost-effective strategy for managing ventricular tachyarrhythmias in patients with ICDs. The ICER remains below the willingness-to-pay threshold in both deterministic and probabilistic sensitivity analyses, demonstrating the robustness of the findings.

## Graphical abstract

**Figure:**
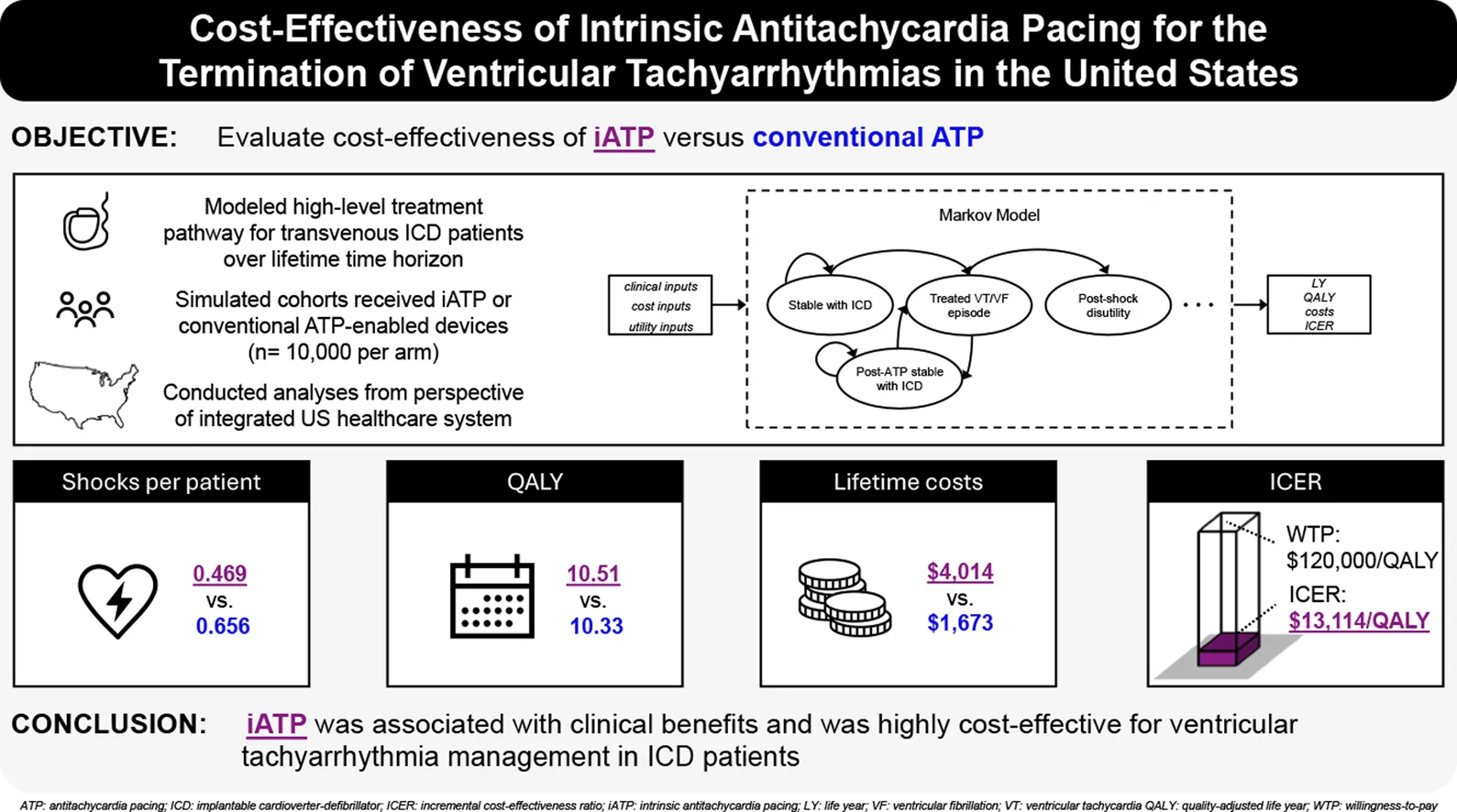

**Key Findings**

▪ In a U.S. lifetime cost-effectiveness model, a novel antitachycardia pacing algorithm (iATP) was cost-effective compared to conventional ATP, with an incremental cost-effectiveness ratio of $13,114 per quality-adjusted life year which is below the willingness-to-pay threshold proposed by physician society guidelines.
▪ The number of shocked episodes estimated by the model was lower for iATP compared to conventional ATP over a lifetime horizon, resulting in improvements in both life years and quality of life relative to conventional ATP despite higher initial device acquisition costs.
▪ The economic value of iATP was driven by reductions in shock-related healthcare utilization and shock-associated quality of life reduction and mortality risk.
▪ The cost-effectiveness findings were consistent across deterministic and probabilistic sensitivity analyses, supporting the robustness of the results.

## Introduction

Ventricular tachycardia (VT) is a severe cardiac arrhythmia that can lead to hemodynamic instability, syncope, or sudden cardiac death if not promptly treated.^1^^,^^2^ VT and ventricular fibrillation (VF) contribute significantly to cardiovascular disease burden worldwide, particularly among patients with structural heart disease such as myocardial infarction or heart failure.^3^^,^^4^

Antitachycardia pacing (ATP) therapy has become an important treatment modality for managing VT in patients with implantable cardioverter-defibrillators (ICDs) with or without cardiac resynchronization therapy.^5^ ATP works by delivering rapid electrical impulses to interrupt the reentrant circuits that are the substrate for VT and restore normal rhythm without requiring ICD shocks, which are painful, are psychologically distressing for some patients, and are associated with an increased risk of mortality.^6^, ^7^, ^8^, ^9^, ^10^, ^11^, ^12^, ^13^, ^14^, ^15^ Despite its promise, the success rate of ATP varies, and ICD shocks are sometimes still required to terminate episodes.^8^^,^^10^^,^^16^, ^17^, ^18^, ^19^, ^20^, ^21^, ^22^ Variability in patient response to ATP highlights the need for more advanced and tailored therapies to optimize VT management and improve patient outcomes.

The intrinsic antitachycardia pacing (iATP) algorithm offers a more individualized approach and represents the first meaningful innovation since the advent of conventional ATP in the early 1990s.^23^ Unlike conventional ATP, which relies on fixed preprogrammed algorithms, iATP analyzes the response to each unsuccessful pacing attempt and automatically adjusts the subsequent pacing strategy to maximize the likelihood of arrhythmia termination.^24^^,^^25^ This approach aims to increase therapy precision and effectiveness, potentially enhancing VT termination while minimizing unnecessary shocks. Several case reports, simulation-based studies, registries, and remote monitoring studies have highlighted the clinical benefits of iATP, establishing it as an improvement over conventional ATP therapy.^25^, ^26^, ^27^, ^28^, ^29^, ^30^, ^31^, ^32^, ^33^

The financial burden of managing VT is substantial. Research shows that ICD shocks are associated with significantly higher health care utilization and costs.^34^^,^^35^ ICD shocks trigger a cascade of health care events, including cardiac procedures such as echocardiography, electrophysiology studies, and other cardiac interventions, and lead revisions.^34^^,^^35^ The economic impact of shock episodes is substantial, with cost estimates (gathered from 2011 to 2016) ranging approximately from $2000 to $19,000 per shock event.^35^, ^36^, ^37^

Although iATP-capable devices typically have higher acquisition costs than do conventional ATP devices, this may be offset by reduced health care utilization because of fewer shocks. However, the detailed economic implications of adopting this technology have not been reported. This question is of particular relevance to the US health care system, in which widespread use of ICDs, and a growing shift toward value-based reimbursement heighten the importance of understanding the cost-effectiveness of novel device technologies. Therefore, the objective of this study was to evaluate the cost-effectiveness of iATP versus conventional ATP.

## Methods

### Model overview

A simulation model was developed of the high-level treatment pathway for patients implanted with a transvenous ICD (with or without cardiac resynchronization therapy capability) to combine observed inputs and extrapolations into a lifetime time horizon. It was used to estimate the costs, years of survival (life-years [LYs]), quality-adjusted life-years (QALYs), and incremental cost-effectiveness ratio (ICER) of iATP vs conventional ATP for the termination of VT episodes. All inputs to the model were derived from published literature, and no additional data were collected for the purposes of this study.

For each treatment arm, costs and outcomes were aggregated in a consistent manner on the basis of a series of decisions and events represented in the model structure. The model considered all costs and health effects from the perspective of an integrated health system (eg, Kaiser Permanente), and all costs and benefits were discounted at a rate of 3% per year^38^ to account for the time value of money.

### Model structure

**Figure 1:**
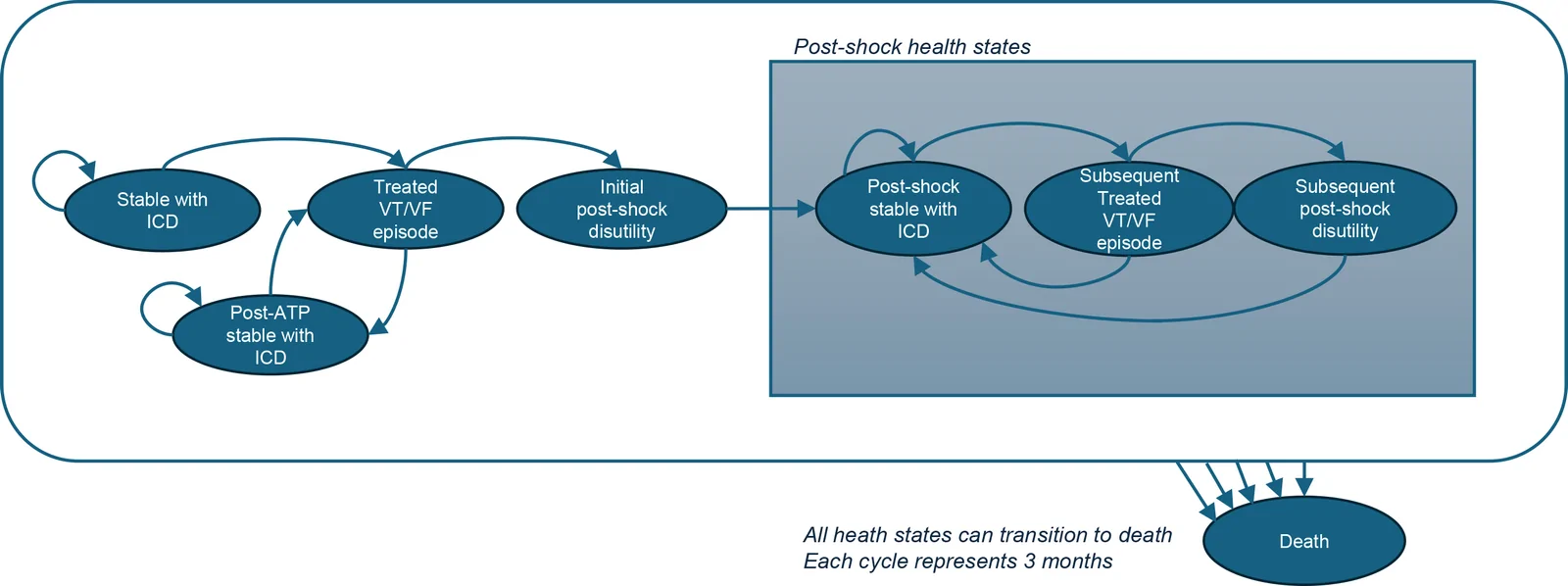
Markov model diagram. A Markov model showing the simulation of the time course of patients receiving an ICD with either iATP or conventional ATP capability. Ovals represent model states; *arrows* indicate transitions between states. For detailed model information, refer to Methods and Supplemental Data 1. ATP = antitachycardia pacing; iATP = intrinsic antitachycardia pacing; ICD = implantable cardioverter-defibrillator; VT/VF = ventricular tachycardia/ventricular fibrillation.

A Markov model was created to evaluate the clinical and economic implications of implanting patients with devices featuring iATP and conventional ATP (Figure 1). The model used 3-month cycles and comprised 8 health states (Supplemental Data 1). Patients entered the model in the “Stable with ICD” health state and received either iATP- or conventional ATP–enabled devices.

Patients remained in the stable state or transitioned to a “Treated VT/VF Episode” state. When in a VT/VF episode, the arrhythmia was terminated by either iATP or conventional ATP, or the patient received an ICD shock if either form of ATP failed to terminate the episode. If the VT/VF episode was resolved by ATP/iATP, the patient transitioned to the “Post-ATP stable with ICD” health state, in which the risk of experiencing recurrent VT events increases compared with the initial stable state. Patients who received a shock entered the “Initial post-shock disutility” state, where they incurred shock-related costs, decreased utility, and increased mortality risk.

After this period of disutility, patients entered the “Post-shock stable with ICD” state, where they faced an increased risk of subsequent VT/VF episodes compared with the initial stable state. The model also included “Subsequent treated VT/VF episode” and “Subsequent post-shock disutility” health states to account for patients who experienced multiple VT/VF events. Patients could experience mortality at any time in the model (on the basis of age- and gender-associated population mortality risk), transitioning to the state called “Death,” where no further clinical or economic data are accrued.

### Clinical inputs

The base-case analysis used data on VT/VF episode occurrence and the effectiveness of ATP and iATP in terminating these episodes without progression to ICD shocks. The time-varying probability of a first VT/VF episode occurring in a specific model cycle was derived to match the time to appropriate therapy for all patients in a recent real-world analysis with 10 years of ICD device data,^39^ extrapolated to later years using a Weibull parameterization (Supplemental Data 2). For subsequent VT/VF episodes after an initial shock, the probability of episode occurrence was derived from a report specifically on the risk of subsequent VT/VF episodes from a pooled analysis of 5 large ICD trials.^40^ The conditional probabilities that a shock would occur, given a treated VT/VF episode were drawn from a postapproval registry of devices with and without iATP.^33^

Age and gender inputs for the model were derived from a weighted average of patient characteristics reported in contemporary ICD trials (Supplemental Data 3). The risk of death associated with ICD shocks was derived from a meta-analysis of 5 studies,^41^, ^42^, ^43^, ^44^, ^45^ which estimated a hazard ratio of 2.55 (95% confidence interval 1.60–4.05) for mortality after a first appropriate shock (Supplemental Data 4). Noncardiac mortality was based on age- and gender-specific general population mortality derived from national life tables.^46^ All clinical inputs and associated references are listed in Table 1.

**Table 1:** Summary of the base-case input parameters of the model, including clinical, cost, and utility values with input sources

Model parameter	Base-case value	Source
Age	64.5 y	Pooled analysis (Supplemental Data 3)
Male gender	81%	Pooled analysis (Supplemental Data 3)
Initial VT/VF episode probability	Time varying	Curtis et al^39^ (Supplemental Data 2)
Subsequent VT/VF episode probability	0.108	Wang et al^40^
Shock probability given a VT/VF episode, iATP	0.165	Yee et al^33^
Shock probability given a VT/VF episode, ATP	0.245	Yee et al^33^
Mortality risk after shock, hazard ratio	2.55	Pooled analysis (Supplemental Data 4)
Background mortality risk	Time varying	Arias et al^46^
Utility—stable with ICD	0.805	Analysis of PainFree SST study data (Supplemental Data 6)
Utility—postshock	0.747	Analysis of PainFree SST study data (Supplemental Data 6)
Incremental cost of iATP	$1550	Manufacturer
Shock HCU cost	$7505	Turakhia et al^35^ (adjusted to 2024)
Device longevity	10 y	Device labeling (Supplemental Data 5)
Discount rate	3.0%	Standard assumption^38^

ATP = antitachycardia pacing; HCU = health care utilization; iATP = intrinsic antitachycardia pacing; ICD = implantable cardioverter-defibrillator; VT/VF = ventricular tachycardia/ventricular fibrillation.

### Cost and utility inputs

The model included costs associated with shock-related health care utilization (both inpatient and outpatient) and incremental cost for an ICD with iATP capability. All other costs related to ICD therapy (eg, complications and follow-up) were expected to be equivalent in both arms and therefore excluded. Where necessary, cost inputs were inflated to 2024 dollars using the consumer price index for medical care services.

The timing of device replacements was derived from projected service life estimates from the labeling for devices with iATP therapy capability (Supplemental Data 5). Health care utilization costs after ICD shocks were derived using the methodology described by Turakhia et al,^35^ which identified shock-related health care utilization through primary diagnoses for inpatient hospitalizations or any relevant diagnosis for outpatient services. The incremental cost of an ICD with iATP capability was obtained from the manufacturer’s average hospital contract price and was applied at initial implantation and at replacement. All cost input parameters are reported in Table 1.

Health state utility values were derived from an analysis of EuroQol 5-Dimension quality-of-life data collected in the PainFree SST clinical trial^12^ and adjusted using US-specific preference weights.^47^ The analysis revealed that the disutility after a shock was temporary and recovered to normal after 1 model cycle (3 months; Supplemental Data 6).

### Analyses

The model simulated cohorts of 10,000 patients with iATP and an equal number of patients with conventional ATP (mean age 64.5 years; 81% male), and the cost and benefit results are presented as mean values per patient. The cumulative estimates of costs and clinical effectiveness were accumulated for each treatment arm to calculate the ICER. The ICER was then compared with the US-specific willingness to pay (WTP) threshold of $120,000 per QALY in alignment with physician society practice guidelines.^48^ Deterministic sensitivity analyses (DSAs) were performed to investigate the impact of key assumptions and parameter values used in the base-case analysis, using ranges for each parameter as detailed in Supplemental Data 7. Probabilistic sensitivity analysis (PSA) using Monte Carlo simulation was also performed, with 1000 iterations to produce a distribution of results from the model. Probabilistic distributions were assigned to each input parameter: beta distributions for probabilities and utilities, gamma distributions for costs, and log-normal distributions for hazard ratios. The outcomes were reported using cost-effectiveness scatter plots and cost-effectiveness acceptability curves. The Markov model was developed in Microsoft Excel, and model fitting and extrapolation were done using the scipy.optimize package in Python.^49^ The economic evaluation was conducted and reported in accordance with the Consolidated Health Economic Evaluation Reporting Standards (CHEERS) guidelines.^50^

## Results

### Base-case cost-effectiveness results

The cohort with iATP experienced a mean of 0.32 initial and 0.15 subsequent shock episodes per patient, while the cohort with conventional ATP experienced a mean of 0.38 initial and 0.27 subsequent shock episodes per patient. Use of iATP resulted in 13.06 LYs (17.45 LYs undiscounted), while conventional ATP resulted in 12.84 LYs (17.07 LYs undiscounted). Measured in QALYs, the discounted values from the use of iATP and conventional ATP were 10.51 and 10.33, respectively, resulting in an incremental effectiveness of iATP of 0.18 QALYs. Discounted costs from iATP and conventional ATP were $4014 and $1673, respectively. The ICER for iATP was $13,114 per QALY as compared with conventional ATP, which is below the recommended WTP threshold of $120,000 per QALY.

### Sensitivity analysis results

**Figure 2:**
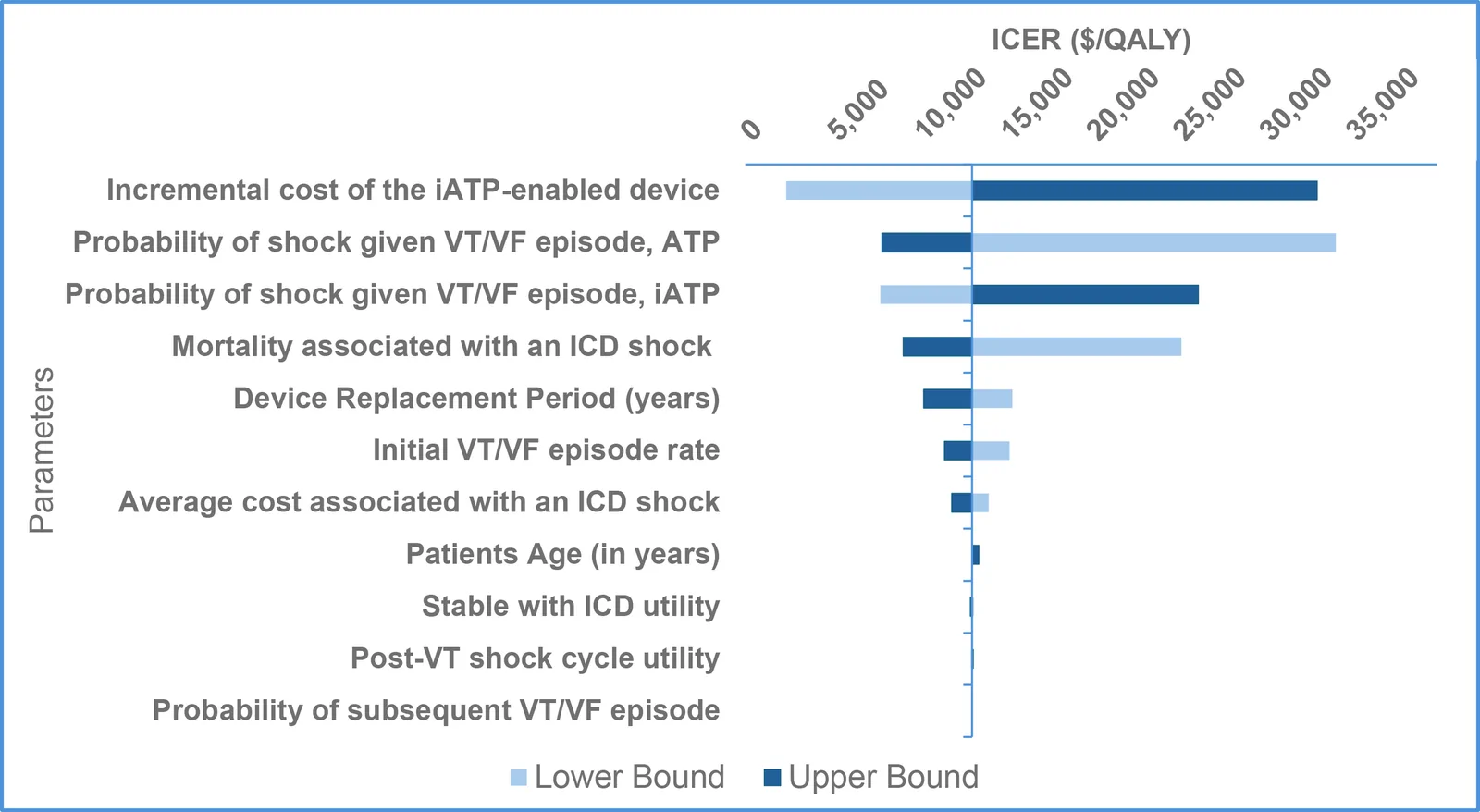
Deterministic sensitivity analysis tornado diagram. The outer ends of the *dark bars* represent ICER values using the upper bound of the relevant model input while holding other inputs at the base-case value. The outer ends of the *light bars* use the lower bound of the relevant model input. ICER = incremental cost-effectiveness ratio; QALY = quality-adjusted life-year; other abbreviations as in Figure 1.

**Figure 3:**
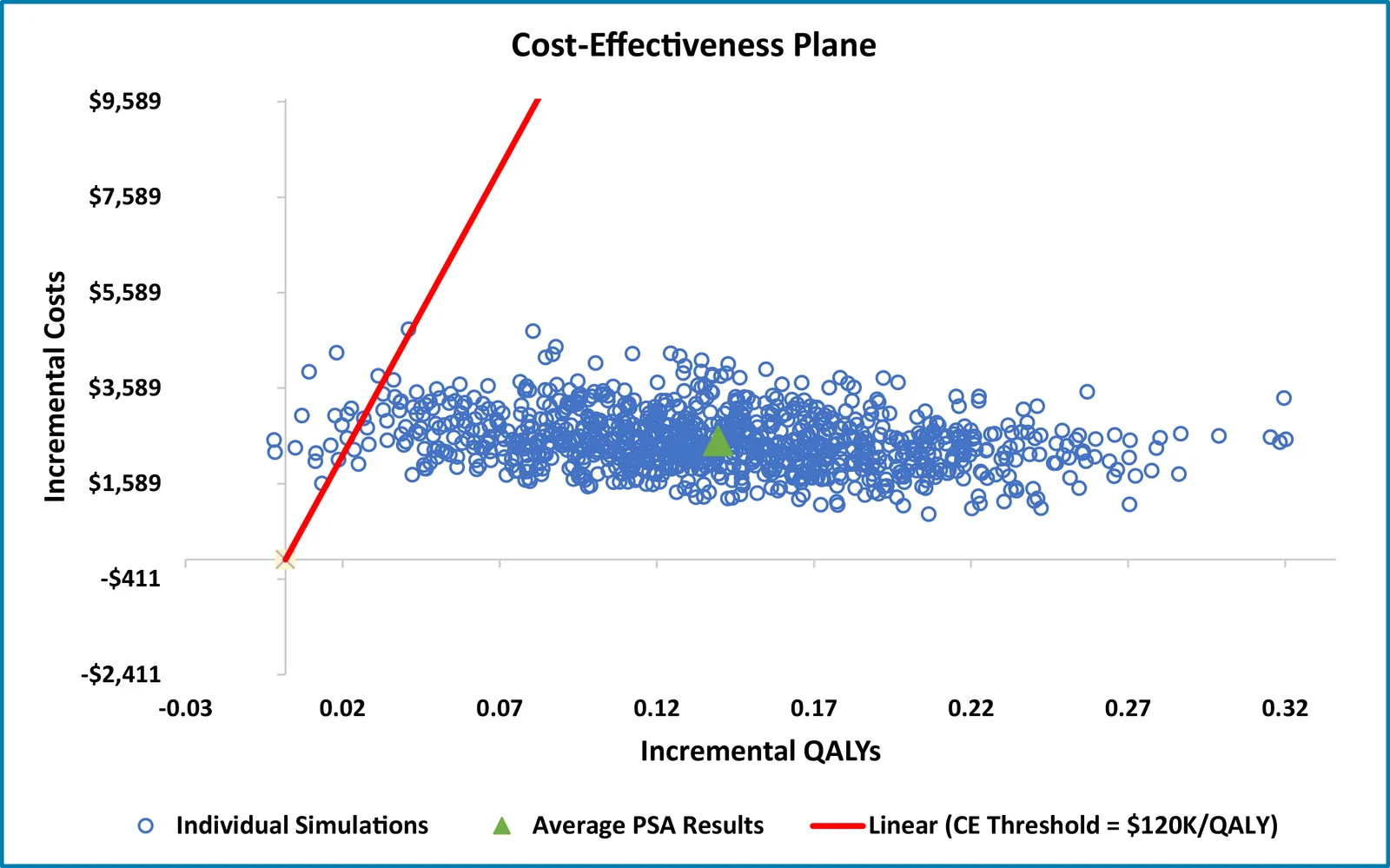
Probabilistic sensitivity analysis scatterplot. The range of ICER given probabilistic variation in model inputs. *Dots* represent individual ICER data points; the red line represents the WTP. All ICER values below and to the right of the *red line* are below the WTP threshold. The green triangle denotes a point composed of the average of all values on the x-axis (Incremental QALYs) and the average of all values on the y-axis (incremental costs). CE = cost effectiveness; PSA = probabilistic sensitivity analysis; WTP = willingness-to-pay; other abbreviations as in Figures 1 and 2.

**Figure 4:**
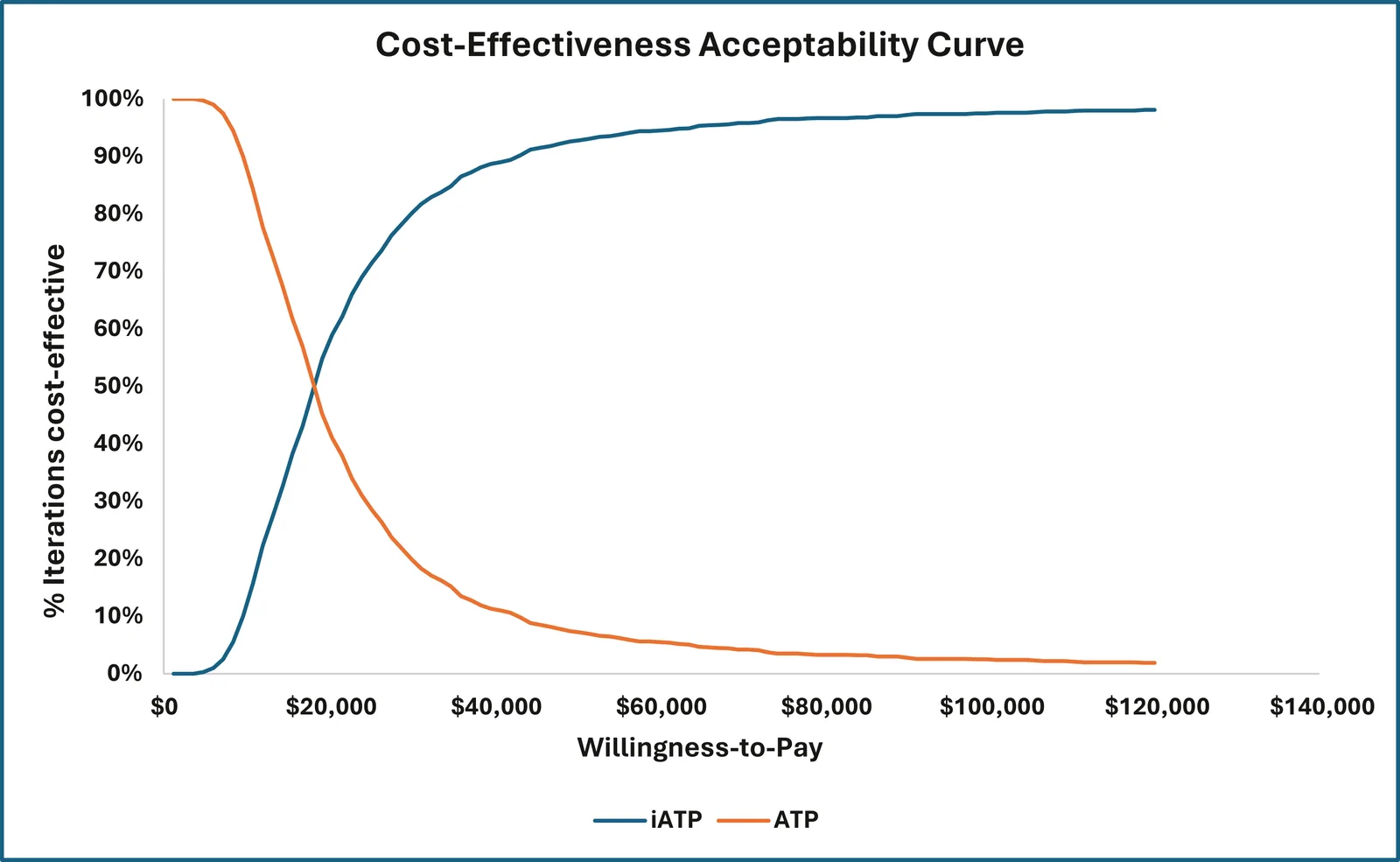
Cost-effectiveness acceptability curves. The curves represent the probability that the incremental cost-effectiveness ratio falls below each willingness-to-pay threshold (x-axis) based on probabilistic sensitivity analysis simulations. The curves cross at 50% on the y-axis, and to the right, all values indicate that iATP cost-effectiveness is more probable. Abbreviations as in Figure 1.

The DSA results showed that the ICER was most sensitive to assumptions related to the incremental cost of iATP, the probability of shock given a treated VT/VF episode, and the postshock mortality (Figure 2); however, the ICER remained below the WTP threshold for all variations in the DSA. In a separate scenario analysis, the ICER remained below the WTP threshold when mortality risk after shock values were ≥1.1. The PSA results had 98% of iterations with the ICER below the WTP threshold (Figure 3). The acceptability curve (Figure 4) shows that the probability that iATP is cost-effective rises above 50% (where the 2 curves cross) at an ICER value of $12,690 per QALY.

## Discussion

Our analysis demonstrates that the use of a novel ATP algorithm (iATP) is associated with an ICER of $13,114, well below the recommended US-specific WTP threshold of $120,000 per QALY. This finding remained robust across DSA and PSA. Collectively, these results indicate that, despite higher upfront costs, iATP provides substantial value to patients by delivering clinically meaningful benefits at a highly favorable cost-effectiveness profile. To our knowledge, no prior economic evaluation has compared iATP with conventional ATP.

The findings of this analysis have their underpinnings in the improved termination efficacy of iATP versus conventional ATP, for which there is a growing body of evidence. An early publication used simulation modeling to demonstrate that the algorithm used to develop iATP was able to terminate VT episodes that were not terminated by conventional ATP.^24^ Another study found that when patients were treated with iATP or conventional ATP in random order, iATP was more effective than additional conventional ATP at terminating episodes after conventional ATP failed.^25^ Two separate postmarket registries of iATP performed indirect comparative analyses of historical data on conventional ATP, and both found iATP to have a significantly higher termination efficacy.^31^^,^^33^

Although ICDs effectively reduce mortality from life-threatening arrhythmias overall, avoiding ICD shocks that can be terminated with pacing therapy is clinically important. As previously discussed, shocks have been shown to adversely affect quality of life, increase health care costs, and are also associated with increased mortality. Shocks may also prompt potentially unnecessary downstream testing with associated procedural risks, as observed in Turakhia et al.^35^ Among patients hospitalized for an appropriate ICD shock, 79.4% underwent coronary angiography, of whom only 6.5% ultimately required percutaneous coronary intervention. These findings further underscore the importance of minimizing ICD shocks and highlight the value of more effective ATP strategies, such as iATP.

This model incorporates an assumption that shocks increase mortality risk and that reducing the number of shocks by using iATP reduces this risk. Although the causal nature of shocks and mortality risk remains uncertain, both the arrhythmic substrate and the occurrence of shocks are likely to contribute to mortality risk,^15^ with the most compelling evidence being the reduction in mortality associated with programming that led to reduced shock burden in the randomized Multicenter Automatic Defibrillator Implantation Trial—Reduce Inappropriate Therapy.^51^ An additional scenario in the sensitivity analysis varied the hazard ratio for mortality after shock from the base-case value down to a neutral effect. The ICER remained below the WTP threshold for all values down to a near-neutral value of 1.1 but was above the WTP threshold at a hazard ratio of 1.0. In addition, the model captures the well-established negative impact of shocks on health-related quality of life, ensuring that both survival and quality-of-life effects contributed to the overall cost-effectiveness results.

There are limitations that should be acknowledged and considered when interpreting the findings of this study. The inputs to the model were largely derived from literature rather than prospective data collection designed specifically for this analysis. Efforts were made to use the most recent and robust sources of data and to use pooled data from multiple sources where available to mitigate this limitation. 1 constraint of a modeling approach is that individual patient characteristics and the effects of all possible interventions (eg, catheter ablation, medical therapy, and device reprogramming) are not accounted for; however, the inputs are based on studies with large samples, and the outputs are calculated over a large cohort estimating average expected results. Costs and benefits were modeled beyond the timeline of direct observations in the literature; however, this is a standard approach in economic modeling and necessary to estimate the lifetime horizon effects. The model does not account for the full costs of ICD therapy such as standard follow-up activity and complications, which are assumed to be equivalent between the comparison arms. Health state utility values were calculated using standard measures of quality of life, which may not fully account for shock-specific patient impacts such as anxiety or depression after shock episodes. This analysis adopts the perspective of an integrated US health system, which may not be directly applicable to other health care settings or payer models. The findings may not generalize to countries with different health care structures, costs, or patient populations.

## Conclusion

iATP was associated with higher initial costs, fewer shocks, and additional QALYs. The result was an ICER that was below the WTP threshold, indicating that iATP is highly cost-effective as an alternative to conventional ATP for the termination of ventricular tachyarrhythmias and avoidance of shocks in patients with ICDs. The ICER remained below the WTP threshold in both DSA and PSA, demonstrating the robustness of the findings.

## Disclosures

Dr Zweibel has served as a consultant and speaker for Medtronic. Dr Dukes has served as a consultant and speaker for Biotronik and received research funding from the company. He has also served as a consultant for Boston Scientific and Johnson & Johnson and received research funding from both companies. In addition, he has served as a speaker for Johnson & Johnson and Medtronic and received research funding from Medtronic. Mr Roberts, Mr Monje, and Dr Mozingo are employees of Medtronic. Mr Holbrook is an employee and shareholder of Medtronic. Dr Curtis has served on advisory boards for Abbott, Janssen Pharmaceuticals, and Medtronic and has received speaker honoraria from Abbott, Medtronic, and Sanofi Aventis.

## Footnotes

Appendix Supplementary data associated with this article can be found in the online version at https://doi.org/10.1016/j.hroo.2026.05.015.
