# Supplementary material for: Cost-effectiveness of a novel antitachycardia pacing algorithm for the termination of ventricular tachyarrhythmias in the United States
Source: Heart Rhythm O2. 2026 Jun 2;7(9):1706–13. doi: 10.1016/j.hroo.2026.05.015 (PMC13594453; doi:10.1016/j.hroo.2026.05.015)
Supplement: Supplementary file 2 [file mmc2.docx]

**SUPPLEMENT**

Contents

[**Supplement 1. Model State Definitions**	2](#_Toc220068566)

[**Supplement 2. VT/VF Episode Rate Validation**	3](#_Toc220068567)

[**Supplement 3. Age and Gender**	5](#_Toc220068568)

[**Supplement 4. Shock Impact on Mortality**	6](#_Toc220068569)

[**Supplement 5. Device Replacement Period**	7](#_Toc220068570)

[**Supplement 6. Utilities**	8](#_Toc220068571)

[**Supplement 7. Sensitivity Analysis Details**	9](#_Toc220068572)

# **Supplement 1. Model State Definitions**

The model employs a cycle length of 3 months and is designed to operate over a lifetime horizon, capturing long-term outcomes and costs. This 3-month cycle length was selected as it appropriately balances clinical relevance with computational efficiency. This cycle length is consistent with typical follow-up patterns for ICD patients, where device interrogations and clinical evaluations often occur quarterly, particularly following shock events.^1, 2^ Three months also represents a meaningful timeframe for capturing post-shock effects on patient quality of life and healthcare utilization, aligning with the duration of utility decrements observed in clinical studies.^3, 4^ Additionally, a 3-month cycle provides enough granularity to model the temporal dynamics of VT/VF episodes and shock occurrences without creating an excessive computational burden or requiring data at a frequency that exceeds what is reliably available from clinical sources.

| **State Name** | **Definition** |
| --- | --- |
| **Stable with ICD** | Patients receive ICD implant with either iATP or conventional ATP therapy but have not yet experienced an appropriately treated VT/VF episode. |
| **Treated VT/VF episode** | Patient experiences an appropriately treated VT/VF episode. This episode may be terminated by the type of ATP therapy assigned to the patient, or it may lead to a shock. |
| **Post-ATP stable with ICD** | This is a state much like “Stable with ICD” but contains patients who have experienced at least one treated VT/VF episode but have not yet received a high energy shock (only ATP). The probability of having a subsequent VT/VF episode is elevated in this state. |
| **Initial post-shock disutility** | This state happens when the patient has been treated with their first high energy shock, and healthcare utilization costs and negative impact on quality of life are accumulated in the model. |
| **Post-shock stable with ICD** | This is a state much like “Stable with ICD” but contains patients who have experienced at least one high energy shock. The probability of death is elevated in this state compared to patients who have never received a shock. In addition, if this was the first VT/VF episode, the probability of having a subsequent VT/VF episode is elevated. |
| **Subsequent treated VT/VF episode** | Patient experiences an appropriately treated VT/VF episode (similar to “Treated VT/VF Episode” above, but for patients who have received at least one shock previously). This episode may be terminated by the type of ATP therapy assigned to the patient, or it may lead to a shock. |
| **Subsequent post-shock disutility** | This state happens when the patient has been treated with any subsequent high energy shock (beyond their first), and healthcare utilization costs and negative impact on quality of life are accumulated in the model. |
| **Death** | Patient has died from any cause and no longer contributes life years or costs to the model. |

# **Supplement 2. VT/VF Episode Rate Validation**

To validate the rate of treated VT/VF episodes in the model, the model was programmed to output data on initial treated VT/VF episodes experienced by simulated patients, along with mortality experienced as a censoring event. This data was plotted in the blue line in the figure below.

Additionally, the annual Kaplan-Meier estimates of time to first treated VT/VF episode experienced from the large, real-world analysis of ICD patients^5^ were added to the plot as black data points. The data from the publication go out to 10 years post ICD implant, and several candidate parameterizations were considered to allow extrapolation of the data beyond the 10-year period (see figure below).

The best fit curve (according to the relative Akaike Information Criterion, see table below) was a Weibull, so this was used to extrapolate the data. By inspection there is reasonable agreement between the publication and the model regarding treated VT/VF episodes over time.

|  | Weibull | LogLogistic | LogNormal | Exponential |
| --- | --- | --- | --- | --- |
| Parameters | 21.0005, 0.597519 | 11.8758, 0.720847 | 2.49484, 2.30848 | 0.0762204 |
| RMSE | 0.003297 | 0.005179 | 0.006557 | 0.047834 |
| R^2 | 0.999426 | 0.998583 | 0.997728 | 0.879089 |
| AIC | -121.726 | -111.788 | -106.597 | -64.880 |

RMSE = root mean square error; R^2 = coefficient of determination; AIC = Akaike Information Criteria

# **Supplement 3. Age and Gender**

Trials that used modern delayed episode detection strategies to reduce overall shocks were included. A weighted average of age and gender was calculated for inclusion in the model. These were incorporated into the model to ensure that age-specific mortality rates and other age-dependent parameters accurately reflected the demographic profile of patients typically receiving these devices.

| Trials | Authors | N | Avg. or Median age | % Male |
| --- | --- | --- | --- | --- |
| MADIT-II^6^ | Moss et al. 2002 | 742 | 63 | 84.0% |
| MADIT-RIT (Delayed Therapy Arm)^7^ | Moss et al. 2012 | 486 | 62 | 75.0% |
| RAID^8^ | Zareba et al. 2018 | 1012 | 64 | 82.0% |
| ADVANCE III^9^ | Gasparini et al. 2013 | 1920 | 65 | 84.0% |
| PREPARE^10^ | Wilkoff et al. 2008 | 700 | 65 | 79.0% |
| PRAETORIAN (TV-ICD Arm)^11^ | Knops et al. 2020 | 423 | 64 | 83.0% |
| PainFree SST^12^ | Auricchio et al. 2015 | 2770 | 65 | 79.0% |
| Weighted Average |  |  | 64.5 | 81.0% |

# **Supplement 4. Shock Impact on Mortality**

To account for the increased mortality risk associated with appropriate ICD shocks, a meta-analysis was conducted using data from five studies: Ruwald et al. (2014)^13^, Proietti et al. (2015)^14^, Sun et al. (2016)^15^, MacIntyre et al. (2019)^16^, and Aktas et al. (2021)^17^. These studies were selected based on their relevance, quality, and recency, providing a comprehensive view of the mortality risk associated with ICD shocks. The hazard ratios and confidence intervals from these studies were pooled using established methods.^18, 19^ The meta-analysis of the hazard ratios employed a random effects model with inverse variance weighting, and the standard normal quantile methods were used to compute the confidence intervals.^20^ This was estimated using the metagen function from the meta R package.^21^ The pooled hazard ratio for mortality following a first appropriate shock is estimated to be 2.55 (95% CI: 1.60 – 4.05). This indicates that patients who experience an appropriate shock are associated with a significantly higher risk of death compared to those who do not. The output from the R analysis is included below.

```
Review:     Appropriate Shocks in ICD patients

                           HR            95%-CI %W(random)
Proietti et al., 2015  2.9500 [2.1187;  4.1075]       21.1
Sun et al., 2016       2.1100 [1.5071;  2.9542]       21.1
MacIntyre et al., 2019 1.2300 [0.8426;  1.7955]       20.5
Aktas et al., 2021     2.7800 [2.1434;  3.6057]       22.0
Ruwald et al., 2014    6.3200 [3.1314; 12.7554]       15.3

Number of studies: k = 5
```

```
                         HR           95%-CI    z  p-value
Random effects model 2.5500 [1.6048; 4.0519] 3.96 < 0.0001

Quantifying heterogeneity (with 95%-CIs):
 tau^2 = 0.2357 [0.0529; 2.8263]; tau = 0.4855 [0.2299; 1.6812]
 I^2 = 82.3% [59.3%; 92.3%]; H = 2.38 [1.57; 3.60]

Test of heterogeneity:
     Q d.f. p-value
 22.58    4  0.0002

Details of meta-analysis methods:
- Inverse variance method
- Restricted maximum-likelihood estimator for tau^2
- Q-Profile method for confidence interval of tau^2 and tau
- Calculation of I^2 based on Q
```

# **Supplement 5. Device Replacement Period**

Projected service life estimates under a variety of use conditions were extracted from the device manuals for current iATP capable devices and included in the table below. A value of 10 years with a standard error of 0.4 years was empirically selected as representative of the variability of longevity given changes in the major factors that impact device longevity.

| Device | RA Amplitude/ Pace % | RV Amplitude/ Pace % | LV Amplitude/Pace % | Impedance (ohms) | Projected Service Life (years) |
| --- | --- | --- | --- | --- | --- |
| Cobalt XT VR | NA | 2.0 V/0% | NA | 500 | 13.6 |
|  |  | 2.0 V/0% |  | 437 | 13.6 |
|  |  | 2.0 V/100% |  | 500 | 11.9 |
|  |  | 2.0 V/100% |  | 500 | 11.2 |
| Cobalt XT DR | 1.5 V/0% | 2.0 V/0% | NA | 500 | 12.3 |
|  | 1.5 V/35% | 2.0 V/0% |  | RA 449/  RV 428 | 11.9 |
|  | 1.5 V/100% | 2.0 V/0% |  | 500 | 11.4 |
|  | 2.5 V/15% | 2.5 V/100% |  | 500 | 9.8 |
| Cobalt HF CRT-D | 1.5 V/0% | 2.0 V/42% | 1.75 V/100% | 500 | 10.1 |
|  | 1.5 V/13% | 2.0 V/42% | 1.75 V/100% | RA 435  RV 430  LV 568 | 10.1 |
|  | 1.5 V/100% | 2.0 V/42% | 1.75 V/100% | 500 | 9.8 |
|  | 2.5 V/15% | 2.5 V/<5% | 2.5 V/100% | 500/600 | 9.4/9.7 |
|  | 2.5 V/15% | 2.5 V/50% | 2.5 V/100% | 500/600 | 8.8/9.2 |
|  | 2.5 V/15% | 2.5 V/100% | 2.5 V/100% | 500/600 | 8.3/8.7 |

Abbreviations: RA= Right Atrial; RV=Right Ventricular; LV=Left Ventricular; VR=single chamber ICD; DR=dual chamber ICD; CRT-D=ICD with cardiac resynchronization therapy

All manuals accessed 14-Jan-2026:

Cobalt™ XT VR MRI SureScan™ DVPA2D1

<https://www.medtronic.com/content/dam/emanuals/crdm/M046408C001A_view.pdf>

Cobalt™ XT DR MRI SureScan™ DDPA2D1

<https://www.medtronic.com/content/dam/emanuals/crdm/M046406C001A_view.pdf>

Cobalt™ HF CRT-D MRI SureScan™ DTPB2D1

<https://www.medtronic.com/content/dam/emanuals/crdm/M046410C001A_view.pdf>

# **Supplement 6. Utilities**

The model incorporates health state utility values derived from a secondary analysis of the PainFree SST clinical trial.^3^ While literature has documented the psychological impacts of ICD shocks, there is limited evidence that translates this into utility values suitable for cost-effectiveness analysis.

To address this gap, raw de‑identified patient level EQ-5D data from PainFree SST were reanalyzed to derive utilities appropriate for cost-effectiveness analysis. All individual study consent forms were reviewed to ensure the necessary permissions for using the data in other research. The study collected EQ-5D data at baseline, at shock events, and monthly for six months afterward, allowing us to characterize both the magnitude and duration of shock impacts on quality of life. Following a methodology similar to that used in the WRAP-IT study's analysis of device infection impact on quality of life^22^, we evaluated utility changes longitudinally across multiple country cohorts.

The objective of the analysis was to estimate US health state utility values over time following ICD shocks, using patient-reported quality of life (QoL) data. The EQ-5D data was extracted from a SAS dataset and prepared in R by loading necessary packages for manipulation, statistical summaries, and document formatting. US utility weights were drawn from the EQ-5D website (<https://euroqol.org/wp-content/uploads/2024/01/US_reversecrosswalk_SAS.txt>). Health states were defined based on time since device implantation or shock, covering stable periods and post-shock cycles (cycles for the model represent 3-month periods). Utility values were calculated for each health state, including mean values, standard errors, and 95% confidence intervals, rounded to three significant digits.

US Utilities per Model State

| **State** | **N = 6,450**^1^ | **95% CI** |
| --- | --- | --- |
| Stable with ICD | 0.805 (0.002) | 0.800, 0.810 |
| Post-Shock Cycle 1 | 0.747 (0.012) | 0.724, 0.771 |
| Post-Shock Cycles 2-4 | 0.805 (0.012) | 0.782, 0.827 |
| ^1^Mean (Std. Err) | | |
| Abbreviation: CI = Confidence Interval | | |

Since the confidence interval for the first post-shock cycle does not overlap with the Stable with ICD state, a disutility was used in the model for this state. Since the confidence intervals for subsequent states post-implant strongly overlap with the Stable with ICD state, it was assumed that no disutilities should be applied beyond the first cycle.

# **Supplement 7. Sensitivity Analysis Details**

The Deterministic Sensitivity Analysis was performed by varying a single model input to its lower and upper values while holding all other model inputs at the base case value and recording the ICER in each case. The Probabilistic Sensitivity Analysis repeated the ICER calculation over 1,000 iterations, with all model inputs being selected randomly from a probability distribution described by the mean and standard error of each input.

| Model Parameter | Base Case Value | SE (PSA) | Distribution | Lower Value (DSA) | Upper Value (DSA) | Rationale |
| --- | --- | --- | --- | --- | --- | --- |
| Age | 64.5 | 2.58 | Normal | 59.4 | 69.5 | Encompass 95% CI given mean and SE |
| Initial VT/VF Episode | Time Varying | NA | NA | -20% | +20% | Empirically chosen to represent a reasonable range |
| Subsequent VT/VF Episode | 0.108 | 0.0008 | Beta | 10.66% | 10.97% | Encompass 95% CI from subsequent episode analysis |
| Shock Given VT/VF Episode, iATP | 0.165 | 0.017 | Beta | 13.2% | 19.8% | +/- 20% of the mean |
| Shock Given VT/VF Episode, ATP | 0.245 | 0.025 | Beta | 19.6% | 29.4% | +/- 20% of the mean |
| Mortality Risk after Shock | 2.55 | 0.237 | Log-Normal | 1.6 | 4.05 | Encompass 95% CI from mortality analysis |
| Utility – Stable with ICD | 0.805 | 0.002 | Beta | 0.800 | 0.810 | Encompass 95% CI from utility analysis |
| Utility – Post Shock | 0.747 | 0.012 | Beta | 0.724 | 0.771 | Encompass 95% CI from utility analysis |
| Incremental Cost of iATP | $1,550 | $310 | Gamma | $500 | $3,500 | Empirically chosen to represent a reasonable range |
| Shock HCU Cost | $7,505 | $689 | Gamma | $2,229 | $4,921 | Encompass 95% CI from cost analysis |
| Device Longevity (Years) | 10 | 0.4 | Normal | 8.3 | 13.6 | Range of values in device labeling |

Abbreviations: PSA= Probabilistic Sensitivity Analysis; DSA=Deterministic Sensitivity Analysis; CI=Confidence Interval; SE=Standard Error

**SUPPLEMENT REFERENCES**

1.	Burri H, Senouf D. Remote monitoring and follow-up of pacemakers and implantable cardioverter defibrillators. EP Europace 2009;11:701-709.

2.	Ferrick AM, Raj SR, Deneke T, et al. 2023 HRS/EHRA/APHRS/LAHRS expert consensus statement on practical management of the remote device clinic. Heart Rhythm 2023;20:e92-e144.

3.	Sears SF, Rosman L, Sasaki S, et al. Defibrillator shocks and their effect on objective and subjective patient outcomes: Results of the PainFree SST clinical trial. Heart Rhythm 2018;15:734-740.

4.	Gopinathannair R, Lerew DR, Cross NJ, Sears SF, Brown S, Olshansky B. Longitudinal changes in quality of life following ICD implant and the impact of age, gender, and ICD shocks: observations from the INTRINSIC RV trial. J Interv Card Electrophysiol 2017;48:291-298.

5.	Curtis AB, Auricchio A, Mittal S, et al. Contemporary Implantable Cardioverter-Defibrillator Benefit. JACC: Clin Electrophysiol 2025;11:2238-2247.

6.	Moss AJ, Zareba W, Hall WJ, et al. Prophylactic implantation of a defibrillator in patients with myocardial infarction and reduced ejection fraction. N Engl J Med 2002;346:877-883.

7.	Moss AJ, Schuger C, Beck CA, et al. Reduction in inappropriate therapy and mortality through ICD programming. N Engl J Med 2012;367:2275-2283.

8.	Zareba W, Daubert JP, Beck CA, et al. Ranolazine in High-Risk Patients With Implanted Cardioverter-Defibrillators: The RAID Trial. J Am Coll Cardiol 2018;72:636-645.

9.	Gasparini M, Proclemer A, Klersy C, et al. Effect of Long-Detection Interval vs Standard-Detection Interval for Implantable Cardioverter-Defibrillators on Antitachycardia Pacing and Shock Delivery: The ADVANCE III Randomized Clinical Trial. JAMA 2013;309:1903-1911.

10.	Wilkoff BL, Williamson BD, Stern RS, et al. Strategic programming of detection and therapy parameters in implantable cardioverter-defibrillators reduces shocks in primary prevention patients: results from the PREPARE (Primary Prevention Parameters Evaluation) study. J Am Coll Cardiol 2008;52:541-550.

11.	Knops RE, Olde Nordkamp LRA, Delnoy PHM, et al. Subcutaneous or Transvenous Defibrillator Therapy. N Engl J Med 2020;383:526-536.

12.	Auricchio A, Schloss EJ, Kurita T, et al. Low inappropriate shock rates in patients with single- and dual/triple-chamber implantable cardioverter-defibrillators using a novel suite of detection algorithms: PainFree SST trial primary results. Heart Rhythm 2015;12:926-936.

13.	Ruwald AC, Schuger C, Moss AJ, et al. Mortality reduction in relation to implantable cardioverter defibrillator programming in the Multicenter Automatic Defibrillator Implantation Trial-Reduce Inappropriate Therapy (MADIT-RIT). Circ Arrhythm Electrophysiol 2014;7:785-792.

14.	Proietti R, Labos C, Davis M, et al. A Systematic Review and Meta-analysis of the Association Between Implantable Cardioverter-Defibrillator Shocks and Long-term Mortality. Can J Cardiol 2015;31:270-277.

15.	Sun S, Johnson J, Degroot P, Brown ML, Obel O. Effect of ICD Therapies on Mortality in the OMNI Trial. J Cardiovasc Electrophysiol 2016;27:192-199.

16.	MacIntyre CJ, Sapp JL, Abdelwahab A, et al. The Effect of Shock Burden on Heart Failure and Mortality. CJC Open 2019;1:161-167.

17.	Aktaş MK, Younis A, Zareba W, et al. Survival After Implantable Cardioverter-Defibrillator Shocks. J Am Coll Cardiol 2021;77:2453-2462.

18.	Tierney JF, Stewart LA, Ghersi D, Burdett S, Sydes MR. Practical methods for incorporating summary time-to-event data into meta-analysis. Trials 2007;8:16.

19.	Tudur Smith C. Meta-analysis of time-to-event data. Cochrane Learning Live. University of Liverpool, UK: Cochrane; 2018.

20.	DerSimonian R, Laird N. Meta-analysis in clinical trials. Control Clin Trials 1986;7:177-188.

21.	Balduzzi S, Rücker G, Schwarzer G. How to perform a meta-analysis with R: a practical tutorial. Evid Based Ment Health 2019;22:153-160.

22.	Wilkoff BL, Boriani G, Mittal S, et al. Impact of Cardiac Implantable Electronic Device Infection: A Clinical and Economic Analysis of the WRAP-IT Trial. Circ Arrhythm Electrophysiol 2020;13:e008280.
